# Simplified Matrix Sentence Test for Pediatric Cochlear Implant Fitting: Single Institution Experience

**DOI:** 10.3390/audiolres15050117

**Published:** 2025-09-16

**Authors:** Giulia Parolin, Carmela Morizzi, Nader Nassif, Maria Grazia Barezzani, Luca Oscar Redaelli de Zinis

**Affiliations:** 1 Department of Otolaryngology-Head and Neck Surgery, ASST Spedali Civili, University of Brescia, 25100 Brescia, Italy; 2 Department of Pediatric Audiology and Phoniatrics, ASST Spedali Civili, University of Brescia, 25100 Brescia, Italy; 3 Department of Pediatric Otolaryngology, ASST Spedali Civili, University of Brescia, 25100 Brescia, Italy

**Keywords:** matrix sentence test, cochlear implants, children

## Abstract

**Background/Objectives**: The Matrix Sentence Test is an audiological evaluation that quantifies the signal-to-noise ratio, expressed in decibels, at which the patient comprehends 50% of the words of a random sentence heard in noise. It is an effective and reliable tool for cochlear implant fitting and follow-up in both adults and children, demonstrating reliability upon repeated administration. A simplified model of the Matrix Sentence Test can be used in children. This study had two main objectives: first, to evaluate the Simplified Matrix Sentence Test for objectively estimating post-fitting CI performance; and second, to assess the influence of various demographic and device-related variables on the results. The variables of interest included gender, manufacturer, placement, microphone position, array position, score in pre-fitting speech audiometry in quiet, age at first implantation, age at test administration, and the interval between the first implant and the test administration. **Methods**: A retrospective study of pediatric patients with cochlear implants was performed. The inclusion criteria were patients aged 7–18 years, with a minimum of two years of cochlear implantation, adequate Italian language proficiency, and regular follow-up attendance. The subjects were administered the Simplified Matrix Sentence Test prior to and following map fitting by an experienced audiologist. **Results**: The study’s sample population included 51 patients who met the established inclusion criteria, with an average age of 13 years. In the preliminary SiIMax test, the average SNR for 50% sentence comprehension in noise was −0.83 ± 1.86 dB. Map adjustments included reductions or increases in comfort and threshold levels, modifications to multiple electrodes, or minor secondary changes. Approximately two days later, the second Simplified Matrix Sentence Test was administered. The average signal-to-noise for sentence comprehension was −2.05 ± 1.73 dB. Univariate and multivariate analyses revealed that no variable had a statistically significant impact on the results. **Conclusions**: The Simplified Matrix Sentence Test demonstrated universal applicability in compliant patients. Post-implant improvement appeared independent of patient demographics and device variables.

## 1. Introduction

A cochlear implant (CI) is an organ replacement device that emulates the cochlea’s function by transmitting sound information to the auditory nerve. CIs consist of two parts: an external speech processor, which collects acoustic information from the environment, and an internal implant, which processes this information into an electrical signal. The signal is transmitted to the implant via a radiofrequency electromagnetic field. The internal device delivers frequency-banded information to an array of electrodes, which stimulate the acoustic nerve fibers innervating the cochlea with electrical impulses according to their tonotopic characteristics.

The channels operate using a dual strategy: first, by corresponding to the frequency bands delivered to cochlear regions responsible for encoding specific frequencies; and second, by imitating the cochlea’s physiological functioning. The base, responsible for encoding high-pitched sounds, functions by simultaneously stimulating multiple neurons to reconfigure the input pattern. Conversely, the apex, responsible for encoding low-pitched sounds, operates through a phase-locking mechanism.

A major challenge for deaf individuals is recognizing verbal communication in noisy environments. Speech audiometry tests, designed to assess intelligibility in competing noise, have been employed to evaluate this phenomenon. The nature of these tests varies depending on the speech material and competitive noise used.

Numerous factors influence the auditory capacity of CI users. Outcomes depend on device limitations and the patient’s pre-implantation clinical profile. The heterogeneity of results can be attributed to factors such as the onset of hearing loss, cognitive ability, active participation in speech therapy rehabilitation, monaural, binaural, or bimodal stimulation, and motivation. Furthermore, some patients may never achieve the ability to perform a competitive noise speech test [1,2,3,4,5].

Speech material and noise can be presented at either fixed or adaptive intensity. ‘Adaptive intensity’ refers to the intensity level of the speech material or competing noise that varies based on the accuracy of the listener’s response. This strategy allows for a highly precise estimation of the intensity required to elicit 50% correct responses (speech reception threshold—SRT50).

Among adaptive speech-in-noise tests, the Matrix Sentence Test (MST) [6] is increasingly popular. The test consists of repetitive sentences, each comprising five random words, presented with background noise. These words are drawn from a total of 50 frequently used words, categorized into 10 subjects, 10 verbs, 10 numerical adjectives, 10 object complements, and 10 qualifying adjectives. The sentences feature both syntactic regularity and a fixed structure. However, they are also semantically unpredictable, with each sentence having 100,000 possible permutations. The verbal message content shows extrinsic phonetic and syntactic redundancy but no semantic redundancy. The speech material is simple, suggesting minimal cognitive load, yet it is randomized, ensuring unpredictability. The lists are balanced [7]. The test can be administered in two formats: ‘closed,’ where the listener indicates the sentence; and ‘open,’ where the listener repeats the sentence [8].

Age is a salient factor in language testing [9,10,11], prompting the development of a streamlined version, known as the Simplified MST (SiIMax), particularly well-suited for children [12]. The SiIMax methodology aligns with the principles of the adult MST but is adapted to the child’s cognitive and developmental level. A 21-word matrix is used, with 14 sentences administered to subjects of various ages. These sentences consist of seven of the ten numbers, adjectives, and nouns utilized in the full version [12].

Both the MST and SiIMax have demonstrated high reliability across various languages, with a test–retest variability of 1 dB observed in adults and children, even with repeated administration [12,13,14,15]. This is a notable finding, given its relevance to the long-term monitoring of subjects with prostheses or CIs, a common practice in clinical settings [12,13,14,15]. This finding suggests that the tool could be an asset for fitting conventional hearing aids and CIs in both adult and pediatric populations [12,13,14,15]. A notable advantage of this tool is its ability to perform in noisy environments, which enhances its practicality and relevance in real-world settings.

This study’s objective is twofold: first, to report variations in SiIMax responses after CI map adjustment and to verify whether parameters typically analyzed to evaluate CI performance influence the results; second, to propose SiIMax as an additional approach usable with existing diagnostic tests to assess mapping adjustment outcomes in pediatric patients.

## 2. Materials and Methods

We performed a retrospective analysis of pediatric CI patients followed up at the Department of Pediatric Audiology, ASST Spedali Civili, University of Brescia, Italy.

The inclusion criteria for the study were as follows:

- Pediatric patients aged 7–18 years;
- Patients with at least one CI;
- Patients with at least two years of active CI use;
- Italianspeaking patients;
- Patients undergoing regular follow-up at our institution;
- Patients who underwent SiIMax before and after dedicated CI mapping by experienced audiologists.

Exclusion criteria included the following:

- Non-compliant patients (e.g., children with cognitive problems or severe syndromes who were unable to comprehend the test rules);
- Foreign patients not exposed to the Italian language for at least two years;
- Pediatric patients younger than seven years of age.

The SiIMax test was conducted in a sound field with participants seated 1 m from the loudspeaker presenting the signal and noise.

The test was administered to the children after appropriate training and prior to audiologic intervention for CI mapping. Patients were subsequently retested with the SiIMax within a 48 h period, using the identical procedure. The primary outcome was the signal-to-noise ratio (SNR) in decibels (dB), at which the patient achieved 50% word comprehension (speech reception threshold—SRT50).

CI mapping interventions consisted of minor changes made according to the patient’s subjective comfort. Other changes included reductions or increases in the threshold (T) level. The T level is defined as the minimum level of electrical stimulation that the patient can just perceive, contributing to the delineation of the auditory threshold. This measurement is obtained during fitting by presenting very soft sounds and asking the patient when they first become audible. The comfort (C) level is defined as the highest level of electrical stimulation a patient can comfortably tolerate without sounds becoming unpleasant or painful, delineating the upper limit of audibility for a given sound. Its determination is achieved through patient inquiry, assessing whether sounds are comfortable, too loud, or tolerable. Additionally, multiple modifications were made to several electrodes.

The primary outcome, SiIMax improvement, was coded as binary, defined as a change greater than 1 dB, considering the reported test–retest variability of 1 dB in the literature [12,13,14,15]. Demographic, clinical, and CI-specific data obtained from medical record review were used as explanatory variables. Variables of interest were derived from factors documented in patient medical charts and deemed relevant by the literature for influencing CI performance. These factors included gender, manufacturer, placement, microphone position (temporal bone in single unit and behind-the-ear), array position, pre-fitting speech audiometry quiet score, age at first implantation, age at test administration, and the interval between first implant and test administration.

Variables included in the analysis were expressed as mean ± standard deviation, median, interquartile range, range of values, and percentages. Univariate analysis was conducted using independent sample *t*-tests for continuous variables, chi-square tests of independence for categorical variables, and Fisher’s exact test when expected cell counts were less than five. Variables included in the analysis were expressed as mean, standard deviation (SD) median, interquartile range (IQR), range of values, and percentages. Binomial logistic regression was used to assess the combined impact of all predictors on improvement. The model’s fit was evaluated using several methods, including residual deviance, Akaike’s Information Criterion, McFadden pseudo-R^2^, and the Likelihood Ratio Test. For all analyses, statistical significance was set at α = 0.05. All analyses were performed using Jamovi statistical software (Version 2.6.0.0).

The present study was conducted in accordance with the tenets of the Declaration of Helsinki. Ethical approval was obtained from the local ethics committee (Territorial Ethics Committee Lombardy 6, IRCCS Policlinico San Matteo, Pavia).

## 3. Results

The final sample comprised 51 children who met the inclusion criteria. The mean age at implantation was 32 ± 27 months (range: 4–141 months). The continuous variables included in the study were (means/SDs) age at first implant (log-transformed) (1.39 ± 0.33) (Shapiro–Wilk *p* = 0.664), interval from first implant (10.1 ± 3.05 years), age at SilMax test (12.8 ± 2.7 years), SNR1 (−0.83 ± 1.86), SNR2 (−2.06 ± 1.73), and ΔSNR (1.23 ± 1.48). Table 1 presents a comprehensive overview of the patient demographics and device variables.

Before CI map intervention, all patients achieved at least 80% speech discrimination in quiet at a mean intensity of 35 dB (IQR, 30–35; range, 20–55). Additionally, 37 patients (72.5%) achieved 100% discrimination at a mean intensity of 55 dB (IQR, 40–55; range, 30–70). For patients who did not reach 100% discrimination, the mean score was 87%.

The CI map fitting procedure included the following adjustments: multiple electrode modifications (*n* = 17, 33%), reduced C and T levels (*n* = 15, 29%), increased C and T levels (*n* = 14, 28%), and other minor changes (*n* = 5, 10%). Subsequent analysis of SNR2 scores revealed a marked enhancement compared to initial SNR1 scores [t(50) = 5.91, *p* < 0.001], with a mean difference of 1.23 dB (95% CI 0.88–1.57), suggesting a substantial effect size (Cohen’s d = 0.83).

The continuous variables under consideration were not significantly associated with improvement. The statistical analysis results are reported below: first implant age log [t(49) = −0.81, *p* < 0.5]; interval from first implant [t(49) = 0.89, *p* < 0.4]; and age at SiIMax [t(49) = −0.08, *p* < 1] (Table 2).

**Figure 1:**
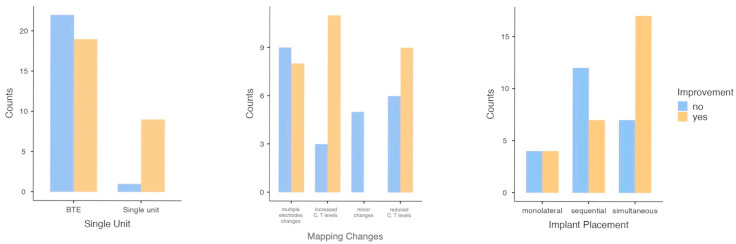
Bar plots of significant and borderline categorical variables at the univariate level.

A notable association with enhancement was identified (Table 1) for single-unit processors (Fisher *p* = 0.01) (Figure 1) and map adjustments (χ^2^ *p* = 0.02) (Figure 1). Implant placement demonstrated a borderline trend (χ^2^ *p* = 0.08) (Figure 1). Variables such as gender, manufacturer, electrode array position, and 100% speech discrimination were not significantly related to improvement (Table 1).

The logistic regression model yielded the following findings: residual deviance = 18.9, Akaike Information Criterion = 52.9, McFadden R^2^ = 0.73, and Likelihood Ratio Test chi-squared = 51.3 (*p* < 0.001). Borderline predictors identified included age at first implant, interval from first implant, and age at SiIMax. The dataset was found to be sparse, indicated by wide or unstable confidence intervals for other predictors. This is consistent with quasi-separation in logistic regression, as illustrated in Table 3.

## 4. Discussion

The most appropriate evaluations to assess CI efficacy and prosthetic gain are those based on speech material, as this represents everyday life most authentically for the patient. Given that a typical day involves exposure to environmental noise, it is more beneficial to conduct tests not only in quiet conditions but also in the presence of competing noise [4,8,14,15].

Among speech-in-noise tests, adaptive tests like the MST have gained popularity in several languages over the past few decades. These tests have demonstrated objective validity and applicability for assessing the hearing performance of hearing-impaired individuals who use hearing aids and CIs [8,16].

The mean SRT50 values in normal hearing adults have been documented for various languages, including Italian, and range from −6.2 ± 0.8 dB SNR to −9.7 ± 0.7 dB SNR [7,17,18]. The Italian MST reported a mean SRT50 of −6.8 ± 0.8 dB SNR for the open response format and −7.3 ± 0.8 dB SNR for the closed response format (training effect was negligible after the third measurement). The test–retest reliability of the Italian matrix test was 0.5 dB for the open response format and 0.6 dB for the closed response format [7].

The results obtained from the MST for adult CI users are expected to be less favorable, with an expected mean SNR of −3.5 ± 1.7 dB, as reported in a Finnish study [18]. The MST has been utilized in adult CI recipients for a variety of purposes. It has been used to compare different map strategies; [19,20] analyze results with reference to the time interval between first and second implants; [21] compare different noise conditions [22] or microphone performance; [23] compare monaural versus bilateral CI performance; [24] evaluate early inpatient rehabilitation after cochlear implantation; [25] assess the subjective and audiological benefit of upgrades [26]. Additionally, it has been used to compare bimodal CI users’ results with age-matched hearing aid users, individuals without subjective hearing loss, and a young normal hearing group [27]. Finally, it helps compare results and subjective listening effort of CI recipients for different sound processing technologies and microphones in adults [28].

The MST results for normal hearing children demonstrated slight variations with age. Puglisi et al. [12] found the average SRT50 to be −5.6 ± 1.2 dB SNR at 5–6 years of age, −5.8 ± 1.2 dB SNR at 7–8 years of age, and −6.6 ± 1.3 dB SNR at 9–10 years of age, using SiIMax. A brief training period yielded high test–retest reliability for all age groups. As in adults, worse results are expected in children with CIs: a mean disadvantage of 5.29 dB was reported for children and adolescents [4].

The MST has been used in pediatric populations to compare outcomes with the initial CI alone versus both CIs in subjects who have undergone sequential implantation. The mean SRT50 was 3.9 dB with the first CI and 2.0 dB with both CIs [29].

The present study shows the results of the SiIMax in detecting enhancements in speech-in-noise performance in pediatric cochlear implant recipients after mapping adjustments. The substantial enhancement in SNR performance indicates that SiIMax could serve as a possible tool for assessing the functional outcomes of clinical programming adjustments.

Of note, the observed improvement was largely independent of demographic or implant-related characteristics, suggesting that SiIMax could demonstrate robust efficacy across heterogeneous pediatric populations. A recent study has demonstrated the sensitivity of the Matrix Sentence Test to subtle changes in speech-in-noise perception in adolescents, depending on factors such as age of implant and cognitive performance [30]. The findings of our study indicated borderline evidence for an age effect: as the interval between implantation and evaluation increases, the map appears to require less adjustment.

The limitations of the MST procedure reported in the literature, such as learning/training effect, cognitive load, working memory, attention, processing speed, and progressive increase in stress and fatigue (particularly evident in hearing-impaired subjects) [31], did not affect the application of SiIMax in our group of children, as all subjects completed the test without encountering any issues.

The primary limitation of this study is its relatively modest sample size, which, combined with the sparse nature of certain predictor variables, limited the efficacy of multivariate analysis. Moreover, this study did not delve into the long-term stability of the observed improvements or the influence of specific mapping strategies in detail. Finally, significant SNR improvement does not necessarily correspond to clinical substantial benefits. Conducting future multicenter studies with larger cohorts is needed to validate the generalizability of the findings and to explore standardized protocols for SiIMax testing in clinical practice. Additionally, this study’s design had an additional limitation: certain patients were excluded due to comorbidities, including cognitive impairments. These cognitive problems are frequently associated with deafness and often necessitate cochlear implantation.

## 5. Conclusions

The SiIMax test could be a valuable clinical tool for monitoring the functional impact of mapping adjustments in pediatric cochlear implant users. It can detect improvements in speech-in-noise performance, supporting its integration into routine follow-up protocols. Larger prospective studies are needed to confirm its utility and to define evidence-based guidelines for its use.

## Figures and Tables

**Table 1:** Association of demographic and device variables with improvement at SilMax test.

	No (N = 23)	Yes (N = 28)	*p* Value
**Gender**			0.1 ^1^
Male	12 (52.2%)	9 (32.1%)	
Female	11 (47.8%)	19 (67.9%)	
**Implant Placement**			0.08 ^1^
Monolateral	4 (17.4%)	4 (14.3%)	
Sequential	12 (52.2%)	7 (25%)	
Simultaneous	7 (30.4%)	17 (60.7%)	
**Manufacturer**			0.1 ^1^
Advanced Bionics	4 (17.4%)	5 (17.9%)	
Cochlear	17 (73.9%)	14 (50%)	
MEDEL	2 (8.7%)	9 (32.1%)	
**Single Unit**			0.01 ^2^
BTE	22 (95.7%)	19 (67.9%)	
Single unit	1 (4.3%)	9 (32.1%)	
**Array Position**			0.1 ^1^
Lateral wall	4 (17.4%)	12 (42.9%)	
Mid scala	3 (13%)	5 (17.9%)	
Mixed	2 (8.7%)	3 (10.7%)	
Perimodiolar	14 (60.9%)	8 (28.6%)	
**100% Speech Discrimination in Quiet**			0.8 ^1^
No	6 (26.1%)	8 (28.6%)	
Yes	17 (73.9%)	20 (71.4%)	
**Mapping Changes**			0.02 ^1^
Changes on multiple electrodes	9 (39.1%)	8 (28.6%)	
Increased C, T levels	3 (13%)	11 (39.3%)	
Minor changes	5 (21.7%)	0 (0%)	
Reduced C, T levels	6 (26.1%)	9 (32.1%)	

^1^, Pearson’s Chi-squared test; ^2^, Fisher’s exact test; BTE, behind the ear.

**Table 2:** Association of continuous variables with improvement at SilMax test.

					95% Confidence Interval					
	Improvement	N	Mean	SE	Lower	Upper	Median	SD	Minimum	Maximum
**First implant age**	**no**	23	36.23	7.3259	21.03	51.42	28.57	35.134	4.200	143.23
	**yes**	28	29.79	3.7788	22.04	37.55	22.22	19.996	4.500	81.43
**Interval from first implant**	**no**	23	10.54	0.6622	9.17	11.91	10.48	3.176	4.860	16.03
	**yes**	28	9.77	0.5593	8.63	10.92	8.93	2.960	2.863	14.67
**Age at SilMax**	**no**	23	12.77	0.6442	11.43	14.11	13.28	3.089	7.238	18.04
	**yes**	28	12.84	0.4641	11.88	13.79	13.02	2.456	8.737	17.46
**First implant age log**	**no**	23	1.35	0.0603	1.22	1.47	1.34	0.289	0.623	1.85
	**yes**	28	1.43	0.0696	1.28	1.57	1.45	0.368	0.653	2.16

Note. The CI of the mean assumes sample means follow a t-distribution with N − 1 degrees of freedom.

**Table 3:** Binomial logistic regression evaluating the combined effects of all predictors on improvement.

Model Coefficients-Improvement					
Predictor	Estimate	SE	Z	*p*	Odds Ratio
**Intercept**	−54.9500	30,823.9420	−0.00178	0.999	1.37 × 10^−24^
**First Implant Age**	−0.0534	0.0319	−1.67511	0.094	0.94803
**Interval From First Implant**	−1.3974	0.7930	−1.76227	0.078	0.24724
**Age at SilMax**	1.2254	0.6951	1.76281	0.078	3.40549
**Gender:**					
** Female–Male**	7.6755	4.8207	1.59220	0.111	2154.87597
**Implant placement:**					
** Sequential–monolateral**	−34.9339	3936.5609	−0.00887	0.993	6.74 × 10^−16^
** Simultaneous–monolateral**	−6.8233	4.6966	−1.45282	0.146	0.00109
**Manufacturer:**					
** Cochlear–AB**	27.6226	29,232.4385	9.45 × 10^−4^	0.999	9.92 × 10^11^
** MEDEL–AB**	47.3943	29,496.3020	0.00161	0.999	3.83 × 10^20^
**Single unit:**					
** Single unit–BTE**	39.6614	6100.3503	0.00650	0.995	1.68 × 10^17^
**Array position:**					
** Mid scala–Lateral wall**	24.3147	29,232.4376	8.32 × 10^−4^	0.999	3.63 × 10^10^
** Mixed–Lateral wall**	19.9580	3936.5549	0.00507	0.996	4.65 × 10^8^
** Perimodiolar–Lateral wall**	−3.9184	7.4434	−0.52643	0.599	0.01987
**100% speech discrimination in quiet:**					
** yes–no**	−1.4366	2.7976	−0.51352	0.608	0.23773
**Mapping changes:**					
** Increased C, T levels–minor changes**	34.9561	9776.5005	0.00358	0.997	1.52 × 10^15^
** Reduced C, T levels–minor changes**	38.3307	9776.4999	0.00392	0.997	4.43 × 10^16^
** Multiple electrodes changes–minor changes**	32.9251	9776.5003	0.00337	0.997	1.99 × 10^14^

Note. Estimates represent the log odds of “Improvement = yes” vs. “Improvement = no”.

## Data Availability

The data presented in this study are available on request from the corresponding author. Data contained within this article are not available due to privacy issues.
